# Transcriptome profiling of kenaf (*Hibiscus cannabinus L.*) under plumbic stress conditions implies the involvement of NAC transcription factors regulating reactive oxygen species-dependent programmed cell death

**DOI:** 10.7717/peerj.8733

**Published:** 2020-03-10

**Authors:** Xia An, Jie Chen, Guanrong Jin

**Affiliations:** 1 Zhejiang Academy of Agricultural Sciences, Hangzhou, China; 2 Huazhong Agricultural University, Wuhan, China

**Keywords:** Kenaf, Fiber crop, Plumbic stress, NAC, Transcription factors

## Abstract

Heavy metal contamination of soils has become a serious global issue, and bioremediation has been proposed as a potential solution. Kenaf (*Hibiscus cannabinus* L.) is a fast growing, non-woody multipurpose annual plant that is suitable for removing excess heavy metals from soils. However, there has been relatively little research on the kenaf molecular mechanisms induced in response to an exposure to heavy metal stress. Thus, whole kenaf seedlings grown under control (normal) and stress (plumbic treatment) conditions were sampled for transcriptome sequencing. Unigenes generated through the *de novo* assembly of clean reads were functionally annotated based on seven databases. Transcription factor (TF)-coding genes were predicted and the physiological traits of the seedlings were analyzed. A total of 44.57 Gb high-quality sequencing data were obtained, which were assembled into 136,854 unigenes. These unigenes included 1,697 that were regarded as differentially expressed genes (DEGs). A GO enrichment analysis of the DEGs indicated that many of them are related to catalytic activities. Moreover, the DEGs appeared to suggest that numerous KEGG pathways are suppressed (e.g., the photosynthesis-involving pathways) or enhanced (like the flavonoid metabolism pathways) in response to Pb stress. Of the 2,066 predicted TF-coding genes, only 55 were differentially expressed between the control and stressed samples. Further analyses suggested that the plumbic stress treatment induced reactive oxygen species-dependent programmed cell death in the kenaf plants via a process that may be regulated by the differentially expressed NAC TF genes.

## Introduction

Heavy metal contamination has emerged as a common problem worldwide. In China, approximately 20% of the available cultivated land has been contaminated by heavy metals from industrial urban emissions and agricultural practices (Deng et al., 2017), which has decreased the usable land area and restricted the distribution of vegetation. Additionally, the bioaccumulation of heavy metals in plants and animals seriously harms the food chain and human health. Bioremediation, which may be useful for minimizing heavy metal contamination, involves the growth of high biomass plants that can accumulate high concentrations of heavy metals on contaminated land. Thus, selecting and breeding cultivars suitable for bioremediation and characterizing the mechanisms underlying the responses of such plants to contaminated conditions are necessary (Xu et al., 2019).

Kenaf (*Hibiscus cannabinus* L.) is a fast-growing, non-woody multipurpose annual plant species in the family Malvaceae. Kenaf fiber has multiple applications in diverse fields, and is included in textile and packing materials, pulp and paper, composite media, animal feed, as well as in potting, building, and filtration materials, while also being useful for board making and as a source of biomass energy (Ayadi et al., 2011). Several factors have made kenaf the third largest fiber crop, behind only cotton and jute. For example, it is an excellent source of cellulosic fiber, it can be used in numerous fiber-based products, it has ideal physical strength properties for pulp and paper, and the energy consumed and chemical input required for pulping and paper-making processes are lower for kenaf than for other comparable wood fibers (Ayadi et al., 2011). In addition to the importance of kenaf as a valuable fiber crop, it has been proposed as a suitable plant for bioremediation because of its rapid growth, large biomass, stress resistance, and adaptability (Deng et al., 2017). However, little is known regarding how kenaf plants respond to various heavy metal stresses.

Previous studies have investigated the physiological mechanisms mediating kenaf responses to cadmium (Cd) stress (Deng et al., 2017). Among the numerous analyzed physiological indices, the malondialdehyde (MDA) content was positively correlated with the Cd concentration and duration of the stress exposure. In another study, researchers proposed various physiological indices for monitoring the responses of two kenaf cultivars to Cd stress (Li et al., 2013). Similarly, the physiological responses of another two kenaf cultivars to chromium (Cr) stress were determined (Ding et al., 2016). These earlier investigations of the molecular mechanisms of kenaf plants induced by heavy metal stresses were conducted in a relatively low-throughput manner (Li et al., 2013; Niu et al., 2018). Additionally, omics approaches (e.g., transcriptomic or proteomic analyses) were mostly applied to investigate the molecular mechanisms of kenaf plants subjected to drought stress (Niu et al., 2016; An et al., 2018).

In this study, we examined kenaf responses to plumbic stress at the transcriptional level. A total of 44.57 Gb high-quality sequencing data were generated for the control and stressed kenaf seedlings and then de novo assembled into 193,978 transcripts from 136,854 unigenes. These unigenes encode 2,066 transcription factors (TFs) belonging to 53 TF families. Moreover, 55 TF genes from 20 TF families were differentially expressed. Further examination of kenaf seedlings revealed that reactive oxygen species (ROS)-dependent programmed cell death (PCD) was induced in response to plumbic stress, in a process that may be regulated by the differentially expressed NAC (NAM, no apical meristem; ATAF, Arabidopsis transcription activation factor; and CUC, cup-shaped cotyledon) TF genes. Therefore, identifying the differentially expressed NAC TF genes based on the high-throughput data produced in this study may help clarify the molecular mechanisms involved in kenaf responses to plumbic stress.

## Materials and Methods

### Plant materials

Kenaf cultivar H368 was obtained from Professor Defang Li (Institute of Bast Fiber Crops, Chinese Academy of Agricultural Sciences). Plants were grown under a 16-h light (28 ° C)/8-h dark (25 °C) cycle, with a relative humidity close to 60% and a light intensity of 700 µmol m^−2^ s^−1^. A pot culture experiment was completed, with each pot ( eight cm height, seven cm diameter) filled with an equal weight of a soil mixture comprising red soil: humus: vermiculite (2:1:1, v/v/v). When the plants grew to a height of nine cm, they were treated with a lead concentration of 4,000 mg/kg (Pb(NO_3_)_2_ treatment solution). We re-applied 4,000 mg/kg of Pb(NO_3_)_2_ in each pot at one time according to the matrix. The application method was to dissolve Pb(NO_3_)_2_ in 1,000 ml ddH_2_O and then pour into the potted soil. Control plants were treated with the same solution without Pb(NO_3_)_2_. Each treatment was completed with two replicates. Three kenaf seedlings per replicate were collected 24 h later. They were immediately frozen with liquid nitrogen and stored at −80 °C until analyzed.

### RNA extraction, library preparation, and sequencing

Total RNA was extracted from the frozen kenaf samples with the TRIzol reagent (Invitrogen, CA, USA). The RNA quality was checked by gel electrophoresis and with the 2100 Bioanalyzer (Agilent, CA, USA) for subsequent analyses. For each biological replicate, cDNA libraries were constructed (i.e., CK1, CK2, Pb1, and Pb2) and sequenced. Briefly, poly-A mRNA was isolated from the total RNA with Magnetic Oligo (dT) Beads and fragmented. Double-stranded cDNA was synthesized with the SuperScript Double-Stranded cDNA Synthesis kit (Invitrogen) and a random hexamer primer (Illumina). After an end-repair and phosphorylation step with T4 DNA polymerase, Klenow DNA polymerase, and T4 polynucleotide kinase, the ends of the cDNA fragments were ligated to Illumina paired-end adapters with T4 DNA ligase. The libraries comprising 200 ± 25 bp cDNA fragments were sequenced with the HiSeq X Ten sequencing platform to produce PE150 reads.

### Data assembly and annotation

Raw sequencing data were processed by removing low-quality reads and reads containing adapter or poly-N sequences. The remaining clean reads were used for all downstream analyses. Transcripts were *de novo* assembled with the default parameters of the Trinity software (Grabherr et al., 2011) and then further clustered into unigenes with the Corset software (Davidson & Oshlack, 2014). The unigenes were functionally annotated based on seven databases. Details regarding these databases, software, and parameters are listed in Table S1.

### Quantification of gene expression levels and analysis of differential expression

The gene expression levels of each sample were estimated by mapping the clean reads to the assembled transcriptome with the default parameters of the RSEM (RNA-Seq by Expectation Maximization) software (Li & Dewey, 2011). The mapped read counts were then transformed to FPKM (fragments per kilobase of transcript sequence per million base pairs sequenced) values to evaluate the relative unigene expression levels (Trapnell et al., 2010). The differential expression induced by the control and Pb stress conditions was analyzed with the DESeq R package (version 1.10.1) (Anders & Huber, 2010), with adjusted *p* < 0.05. Enriched gene ontology (GO) terms among the differentially expressed genes (DEGs) were identified with the GOseq R package based on the Wallenius non-central hyper-geometric distribution (Young et al., 2010). Moreover, the enriched Kyoto Encyclopedia of Genes and Genomes (KEGG) pathways were identified with the KOBAS software (Mao et al., 2005). The predicted TF genes based on the Plant Transcription Factor Database (version 5.0) (http://planttfdb.cbi.pku.edu.cn/) were compared with the DEGs to detect the differentially expressed TF genes.

### Quantitative real-time PCR (qRT-PCR)

Total RNA was extracted from whole kenaf seedlings subjected to normal (CK) or Plumbic (Pb) stress conditions with the RNAqueous Total RNA Isolation Kit (Ambion, USA). The RNA was used as the template to synthesize cDNA with the HiScript II Q RT SuperMix for qPCR (Vazyme, China). The cDNA was diluted 10-fold with sterile water and used for a qRT-PCR assay. The *GAPDH* gene identified in the current transcriptome sequencing analysis (Cluster-5711.64302) served as an internal reference control. The qRT-PCR assay was performed with the LightCycler 480II Real-Time PCR Detection System (Roche Ltd, USA). The 20- µL reaction mixture contained 10 µL ChamQ SYBR qPCR Master Mix (Vazyme), 2 µL cDNA template (from approximately 100 ng total RNA), and 0.5 µM forward and reverse primers (Table S2). The amplification parameters were as follows: 95 °C for 30 s; 40 cycles of 95 °C for 10 s and 60 °C for 30 s. Three independent experiments were completed to ensure the qRT-PCR data were reproducible. Relative gene expression levels were calculated according to the 2^−ΔΔ*CT*^ method (Livak & Schmittgen, 2001).

### Examination of physiological traits

Physiological traits of the CK and stressed samples were examined by Comin Biotechnology Co. Ltd. (http://www.cominbio.com). Regarding MDA content measurements, the frozen samples were ground in liquid nitrogen and then homogenized in 10 volumes (v/w) of pre-cooled potassium phosphate buffer (containing Na_2_HPO_4_ and NaH_2_PO_4_; pH 7.0) on ice. Samples were centrifuged at 8, 000 × g for 10 min at 4 °C. A 0.1-mL aliquot of the supernatant was mixed with 0.3 mL 0.5% (w/v) thiobarbituric acid, which was dissolved in 5% (w/v) trichloroacetic acid, and the extract was boiled for 40 min and then immediately cooled in ice. After centrifuging at 12, 000 × g for 10 min, the absorbance of the supernatant was measured at 532 nm. The effect of non-specific turbidity was eliminated by subtracting the absorbance of the supernatant at 600 nm. The MDA content [nmol/g fresh weight (FW)] was calculated with the following formula: 25. 8 × (A_532_ − A_600_)/weight, in which A_532_ and A_600_ are the absorbances at 532 and 600 nm, respectively.

To measure the peroxidase (POD) and catalase (CAT) contents (i.e., antioxidant enzymes), 10 µL supernatant (generated from the pre-cooled potassium phosphate buffer) was mixed with 190 µL working solution, after which the absorbance was measured twice at 470 nm (for POD) or 240 nm (for CAT). The second measurement (A2) was taken 1 min after the first (A1). The working solution for measuring the POD content comprised guaiacol, sodium acetate, and hydrogen peroxide, whereas the working solution for determining the CAT content was the potassium phosphate buffer supplemented with hydrogen peroxide. The POD and CAT contents (U/g FW) were calculated with the following formulae: 40 × (A2 − A1)/weight and 918 × (A1 –A2)/weight, respectively.

To determine the proline (PRO) content, samples were homogenized in a pre-cooled salicyl-sulfonic acid solution, boiled for 10 min, and centrifuged at 10, 000 × g for 10 min at 25 °C. The supernatant was collected and mixed with the working solution comprising ninhydrin, acetic acid, and phosphoric acid. The resulting solution was boiled for 30 min and then cooled. Methylbenzene was added and the solution was vortexed for 30 s. The absorbance of the upper layer of the solution was measured at 520 nm. The PRO content was calculated with the following formula: 38. 4 × (A_520_ + 0.0021)/weight.

### Statistical analyses

Data were analyzed with SPSS software (version 22.0) (SPSS, Chicago, IL, USA). Error bars represent the standard deviation. The significance of the differences in the data for the control and stressed samples was assessed with Student’s *t*-test (with *, **, and *** corresponding to *p* < 0.05, *p* < 0.01, and *p* < 0.001, respectively).

## Results

### Illumina sequencing and assembly

**Figure 1:**
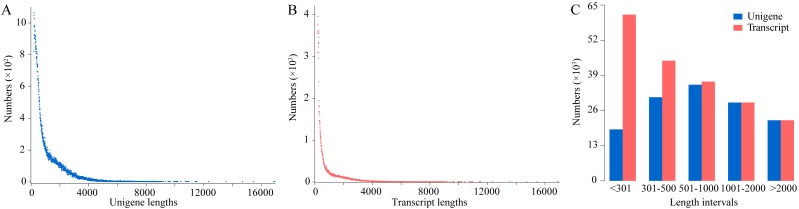
Analysis of transcript and unigene lengths. Length distribution of the assembled transcripts (A) and unigenes (B) are respectively presented, as well as the statistics regarding the number of sequences distributed on different lengths (C).

Young kenaf seedlings under CK or Pb stress conditions were separately collected in duplicate for the construction of four cDNA libraries. A correlation analysis on the transcriptomic output revealed the consistency in the data generated by the replicates of the control (CK1 and CK2) and stressed (Pb1 and Pb2) samples (Fig. S1). The overall sequencing results are presented in Table S3. A total of 297,123,462 clean reads (44.57 Gb) were obtained from our data. The error rates for all four samples were less than 0.1% and the Q30 values exceeded 94.66. These high-quality clean reads were assembled into transcripts with the Trinity software (Grabherr et al., 2011) and were further clustered into unigenes with the Corset software (Davidson & Oshlack, 2014). Consequently, 193,978 transcripts were generated, from which 136,854 unigenes were obtained. The unigenes comprised fewer total nucleotides, but were longer than the transcripts (Table S4 and Fig. 1).

### Gene annotation and function classification

**Figure 2:**
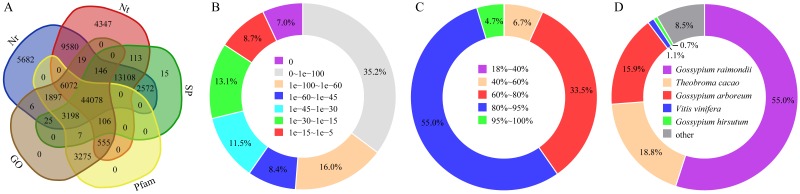
Statistics of unigene annotations. Venn diagram of the unigenes annotated based on five databases (A). Percentage distribution of E-values (B), sequence similarities (C), and top species annotated (D) following a BLAST search of the Nr database with the unigenes as queries. Nr, NCBI non-redundant protein sequence database; Nt, NCBI nucleotide sequence database; Pfam, Protein family database; KOG, euKaryotic Orthologous Groups database; SP, Swiss-Prot database.

The assembled unigenes were functionally annotated based on the NCBI non-redundant protein sequence (Nr) database, the NCBI nucleotide sequence (Nt) database, the Protein family (Pfam) database, the euKaryotic Orthologous Groups (KOG) database, the Swiss-Prot (SP) database, the KEGG database, and the GO database. The number of unigenes annotated with each database is indicated in Table S5. Specifically, 32.21% of the unigenes were commonly annotated with the Nr, Nt, SP, Pfam, and GO databases (Fig. 2A). The *E*-value distribution for the unigenes annotated with the Nr database (i.e., the database with the most annotated unigenes; Table S5) indicated that 78.2% of the annotated unigenes (67,503 of 86,376) had E-values less than 1e−30 (Fig. 2B). Moreover, 93.2% of the unigenes annotated with the Nr database (80,485 of 86,376) matched genes in the database with ≥ 60% sequence similarities (Fig. 2C). Additionally, the unigenes were most similar to genes from ancestral cotton species (*Gossypium raimondii*, 55.0% and *Gossypium arboreum*, 15.9%) (Fig. 2D).

Among the seven databases, functional annotations with the GO, KOG, and KEGG databases enabled the classification of the unigenes in specific categories, thereby providing a more systemic analysis of the transcriptome output. A total of 59,384 unigenes were classified into GO categories, with “cellular process” from the biological process (BP) category, “binding” from the molecular function (MF) category, and “cell” from the cellular component (CC) category identified as the most enriched GO terms in the three main categories (Fig. S2). A smaller proportion of unigenes (32,173, 23.51%) were assigned to KEGG pathways (Fig. S3), with “carbohydrate metabolism” from the metabolism category and “translation” and “folding, sorting and degradation” from the genetic information processing category the top three KEGG pathways. Fewer unigenes (20,553 of 136,854 unigenes) were classified based on the KOG categories. Although all 25 KOG categories were represented, “R: general function prediction only”, “O: posttranslational modification, protein turnover, chaperons”, and “J: translation, ribosomal structure and biogenesis” were the most common (Fig. S4).

For a more specific examination of coding sequences, the transcriptomic data were used as queries for BLAST searches of the Nr and SP databases to predict amino acid sequences. Regarding the unigenes lacking matches with sequences in the Nr or SP databases, their open reading frames were predicted with the ESTScan software (Iseli, Jongeneel & Bucher, 1999). The number of unigenes annotated by a BLAST search (58,846 unigenes) was similar to the number of unigenes whose open reading frame was predicted with ESTScan (56,037 unigenes). However, the peptides encoded by the BLAST-annotated unigenes were longer than the ESTScan-predicted peptides (Fig. S5A). Additionally, most of the BLAST-annotated unigenes harbored only the termination codon. In contrast, there was a more even split between the number of ESTScan-predicted sequences with and without the initiation codon and/or the termination codon (Fig. S5B).

### Differential expression analyses

**Figure 3:**
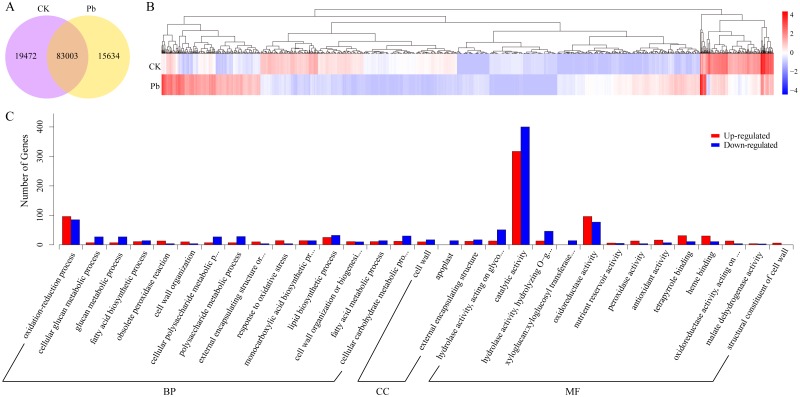
Expression analysis of unigenes. Number of unigenes expressed in CK and Pb stress conditions (A). Diagram of the 1,697 differentially expressed unigenes (DEGs), with blue to red colors representing low to high relative expression levels, respectively (B). Results of the GO enrichment analysis of the up-regulated (red columns) and down-regulated (blue columns) DEGs (C). BP, biological process; CC, cellular component; MF, molecular function.

**Figure 4:**
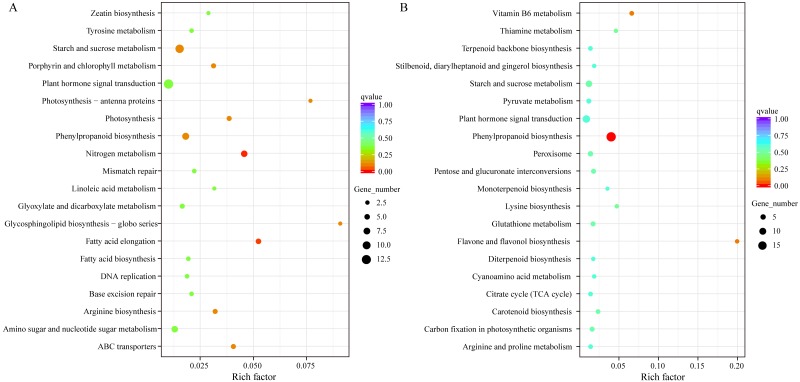
Top 20 enriched KEGG pathways among DEGs. Enriched KEGG pathways among the unigenes with down-regulated expression (A) and up-regulated expression (B) in response to Pb stress.

To preliminarily reveal the molecular mechanisms underlying kenaf seedling responses to Pb stress, the expressed unigenes were compared between the CK and Pb-stressed samples. A total of 102,475 and 98,637 unigenes were respectively expressed in the CK and Pb-stressed samples, with FPKM values exceeding 0.3. Additionally, 83,003 unigenes were expressed in both samples (Fig. 3A). Overall, 1,697 unigenes were identified as differentially expressed between the CK and Pb-stressed samples (Fig. 3B). A GO enrichment analysis of these 1,697 DEGs indicated the three most enriched GO terms were “catalytic activity” and “oxidoreductase activity” from the MF category and “oxidation–reduction process” from the BP category (Fig. 3C). Moreover, the enriched KEGG pathways among the DEGs that were down-regulated in the Pb-stressed samples were “glycosphingolipid biosynthesis”, “photosynthesis”, “fatty acid elongation”, and “nitrogen metabolism” (Fig. 4A). In contrast, “flavone and flavonol biosynthesis”, “vitamin B_6_ metabolism”, and “phenylpropanoid biosynthesis” were the main enriched KEGG pathways among the unigenes with up-regulated expression levels in the Pb-stressed samples (Fig. 4B). The enriched KEGG pathways implied that numerous biological processes that maintain plant growth and development were repressed in kenaf seedlings exposed to Pb stress. The KEGG pathways assigned to the DEGs with up-regulated expression in the Pb-stressed samples may contribute to heavy metal stress responses in kenaf.

**Figure 5:**
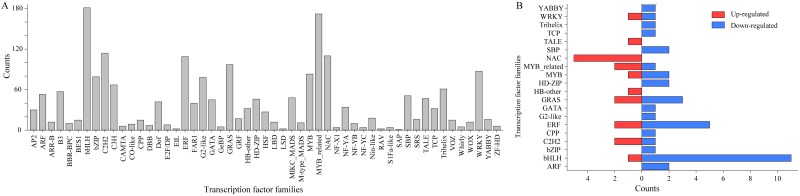
Transcription factors (TFs) examined in the current study. Numbers of predicted TF genes from each TF family among the unigene sequences (A). Differentially expressed TF genes in Pb-stressed compared with CK samples (B).

To probe the possible master regulators of kenaf seedling responses to Pb stress, the DEGs encoding TFs were investigated. The identified unigenes were predicted to include 2,066 TF-coding genes belonging to 53 TF families (Fig. 5A). Among these TF genes, only 55 from 20 TF families had expression levels that were up- or down-regulated in the Pb-stressed samples (relative to the corresponding level in the CK samples) (Fig. 5B). A closer examination of these differentially expressed TF (DE-TF) genes revealed that the down- and up-regulated genes were mainly represented by bHLH (basic helix-loop-helix) and NAC family members, respectively. All five NAC DE-TF genes were up-regulated, whereas 11 of the 12 bHLH DE-TF genes were down-regulated. To verify the reliability of the RNA-seq data, eight randomly selected DE TF genes in Fig. 5B, including all NAC TF genes (Cluster-5711.10506, Cluster-5711.10507, Cluster-5711.10680, Cluster-5711.108716, and Cluster-5711.35745), two ethylene-responsive element-binding factor (ERF) genes (Cluster-5711.107017 and Cluster-5711.107018), and one teosinte branched1/cincinnata/proliferating cell factor (TCP) gene (Cluster-5711.101858), were analyzed by qRT-PCR. The expression levels of all of these TF genes were higher in the Pb-stressed samples than in the CK samples (Fig. S6), which was consistent with the transcriptome sequencing data.

### The NAC TFs in responding the Pb stress

**Figure 6:**
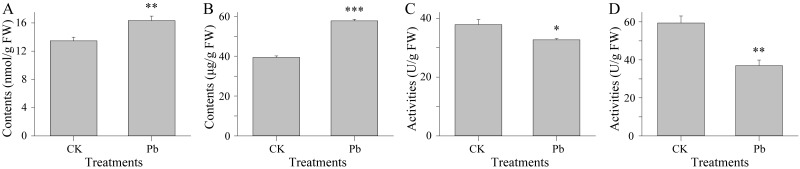
Physiological traits under CK and Pb stress conditions. The MDA (A) and PRO (B) contents as well as the CAT (C) and POD (D) activities are presented on the basis of fresh weight (FW). Error bars represent the standard deviation. Significant differences between the CK and Pb-stressed samples (as determined with Student’s *t*-test) are indicated as follows: ^∗^*p* < 0.05, ^∗∗^*p* < 0.01, and ^∗∗∗^*p* < 0.001.

Only 5 of the 110 NAC TF genes predicted based on our transcriptome data (Fig. 5A) were differentially expressed, with Pb stress up-regulating their expression levels (Fig. 5B), implying they have a regulatory role in the kenaf response to Pb stress. The functional annotation of these five kenaf genes (i.e., Cluster-5711.10506, Cluster-5711.10507, Cluster-5711.10680, Cluster-5711.108716, and Cluster-5711.35745) based on the known functions of the corresponding Arabidopsis orthologs (Table S6) suggested the NAC-involved kenaf response to Pb stress is probably dependent on PCD processes. Meanwhile, the MDA content representing the extent of ROS-induced lipid peroxidation (Ayala, Munoz & Argüelles, 2014) was significantly higher in the Pb-stressed samples than in the CK samples (Fig. 6A; Table S7). Consistent with this finding, the abundance of PRO, which can increase ROS formation (Donald et al., 2001), was also higher under Pb stress conditions than under CK conditions (Fig. 6B; Table S8). Additionally, the activities of the ROS-scavenging enzymes (Paradiso et al., 2016), including POD and CAT, decreased following the Pb treatment (Figs. 6C,6D; Tables S9, S10). These results indicated Pb stress increases ROS levels and the expression of PCD-related NAC TF genes in kenaf.

## Discussion

Kenaf is a broadly used multi-purpose fiber crop (Ayadi et al., 2011). However, there has been minimal fundamental research on this particular species. To date, only a few studies applying RNA-seq approaches have been conducted on kenaf (Chen et al., 2014; Zhang et al., 2015; Li et al., 2016; Li et al., 2017), and only one of them focused on transcriptomic responses under adverse conditions (Li et al., 2017). Thus, relatively little is known about the effects of diverse environmental conditions on the molecular mechanisms in kenaf. The current study involved a transcriptome analysis of whole kenaf seedlings subjected to normal and plumbic stress conditions, with the resulting data potentially useful for elucidating the stress-induced molecular mechanisms at the transcript level. The 1,697 DEGs between the CK and Pb-stressed samples (Fig. 3B) were grouped at various levels (Figs. 3C, 4). The down-regulated pathways (Fig. 4A), including glycosphingolipid biosynthesis and photosynthesis, are required for plant growth. The glycosphingolipids are pivotal molecules for maintaining cellular communication in multicellular organisms (D’Angelo et al., 2013) and photosynthesis is an indispensable source of energy for plants. Thus, plumbic stress appears to suppress critical plant growth processes. In contrast, the up-regulated pathways (Fig. 4B) are likely involved in kenaf responses to adverse conditions. For example, earlier studies proved that diverse flavonoids influence the UV tolerance of rice cultivars and Arabidopsis ecotypes at various latitudes (Tohge & Fernie, 2016; Peng et al., 2017). Additionally, vitamin B_6_ contents have been linked to plant responses to adverse conditions (Bagri et al., 2018; Chandrasekaran & Chun, 2018; Nan et al., 2019). Future studies should focus on the up-regulated pathways as well as the associated unigenes.

Considering the relatively limited molecular research on kenaf, the up-stream regulators of gene expression (i.e., TFs) should be investigated in detail. Accordingly, to identify possible master transcriptional regulators, unigenes encoding TFs were predicted and analyzed (Fig. 5). Many members of various TF families, including MYB (Li, Ng & Fan, 2015), bZIP (Liu & Chu, 2015), bHLH (Yadav & Mani, 2019), TCP (Ding et al., 2019), and NAC (Yuce et al., 2019) TFs are reportedly involved in plant defenses against abiotic stresses. However, these TFs are also important for plant growth (Cheng et al., 2019; Liu et al., 2019; Van Es et al., 2019), which makes identifying the TFs responsive to Pb stress based solely on our transcriptomic data unlikely. Interestingly, the expression levels of all five detected differentially expressed NAC TF genes were up-regulated following the Pb treatment (Fig. 5B), implying these genes may have regulatory roles affecting kenaf responses to Pb stress. Further analyses suggested these NAC TF genes may be involved in PCD (Table S6), which is a highly regulated and organized cell suicide process crucial for removing damaged or infected cells (Petrov et al., 2015). This process is linked to ROS production and accumulation (Nath & Lu, 2015). In plants, ROS are always formed from O_2_ because of the inevitable leakage of electrons during the electron transport activities of chloroplasts, mitochondria, and plasma membranes or as a byproduct of various metabolic pathways localized in different cellular compartments (Rio et al., 2006; Blokhina & Fagerstedt, 2010; Heyno et al., 2011). Adverse environmental conditions (e.g., biotic or abiotic stresses) enhance ROS production in plants because of a disruption in cellular homeostasis (Mittler, 2002; Sharma & Dubey, 2005; Sharma & Dubey, 2007; Maheshwari & Dubey, 2009; Srivastava & Dubey, 2011). In the current study, analyses of MDA and PRO contents and POD and CAT activities (Fig. 6) suggested Pb stress increases ROS levels in kenaf plants. This finding and the observed up-regulated expression of all identified NAC TF genes are possibly associated with PCD (Table S6) and are consistent with the previously established relationship between PCD and ROS (Nath & Lu, 2015). Therefore, we speculate that kenaf plants undergo ROS-dependent PCD in response to plumbic stress, with the up-regulated NAC TF genes tuning PCD.

In previous studies, kenaf plants were subjected to Cd (Li et al., 2013; Deng et al., 2017) and Cr (Niu et al., 2018) stresses. However, these investigations focused on plant physiological traits, but not the underlying molecular mechanisms. Consistent with the findings of these earlier studies, we also observed elevated MDA contents (lipid peroxidation) and suppressed POD activities following an exposure to heavy metal stress, which are phenomena that may be associated with ROS-dependent PCD regulated by several NAC TFs. Future detailed examinations of these NAC TFs may help clarify the molecular mechanisms of kenaf plants subjected to various heavy metal stresses. We may also focus on the three KEGG pathways (flavone and flavonol biosynthesis, vitamin B_6_ metabolism, and phenylpropanoid biosynthesis) that were significantly enriched in the kenaf seedlings under plumbic stress conditions. The observation that these pathways are affected by Pb stress suggests specific metabolites and/or regulatory genes may influence kenaf tolerance to heavy metals. Indeed, vitamin B_6_ metabolites are reportedly responsive to adverse conditions (Bagri et al., 2018; Nan et al., 2019) and the flavonoid-regulating MYB TFs are known to enhance heavy metal stress resistance (Ai et al., 2018; Raldugina et al., 2018). Furthermore, the phenylpropanoid pathway is activated to provide protection against heavy metals via the thickening of physical barriers (Chen et al., 2019). A comprehensive analysis of the up-regulated NAC TF genes and the unigenes involved in the enriched pathways may provide additional insights into the molecular basis of kenaf responses to excessive amounts of heavy metals.

## Conclusions

Our transcriptome sequencing data generated 44.57 Gb clean reads potentially related to kenaf responses to plumbic stress. To probe the possible master regulators of the underlying molecular mechanism, TF genes were predicted and analyzed. On the basis of our transcriptome data combined with analyses of physiological traits, we propose that kenaf seedlings experience ROS-dependent PCD, which may be regulated by the differentially expressed NAC TF genes. Future studies should characterize the roles of these NAC TFs to provide more detailed insights into the molecular mechanisms mediating kenaf plant responses to plumbic stress.

##  Supplemental Information

10.7717/peerj.8733/supp-1 Figure S1 Correlation coefficients among four samples

10.7717/peerj.8733/supp-2 Figure S2 GO annotation of transcriptome data

10.7717/peerj.8733/supp-3 Figure S3 KEGG annotation of transcriptome data

10.7717/peerj.8733/supp-4 Figure S4 KOG annotation of transcriptome data

10.7717/peerj.8733/supp-5 Figure S5 Predicted peptides based on transcripts Length distribution of the peptides encoded by unigenes annotated via a BLAST search of the Nr database (blue) and the peptides encoded in unigenes whose open reading frame was predicted with ESTScan (red) (A). The last blue and red columns include peptides longer than 1,000 amino acids. Analyses of the presence of initiation and/or stop codons in the unigenes encoding the peptides in the blue and red columns (B).

10.7717/peerj.8733/supp-6 Figure S6 Experimental validation of the relative expression levels of randomly selected unigenes Eight unigenes encoding transcription factors (TFs) were randomly selected, including five NAC TF genes (A–E, corresponding to Cluster-5711.10506, Cluster-5711.10507, Cluster-5711.10680, Cluster-5711.108716, and Cluster-5711.35745, respectively), two ERF genes (F and G, corresponding to Cluster-5711.107017 and Cluster-5711.107018, respectively), and one TCP gene (H, Cluster-5711.101858). Error bars represent the standard deviation. Significant differences between the CK and Pb-stressed samples (as determined with Student’s t-test) are indicated as follows: * *p* < 0.05, ** *p* < 0.01, and *** *p* < 0.001.

10.7717/peerj.8733/supp-7 Tables S1–S10 Supplemental Tables 1–10. Brief information of how the unigenes were annotated against the seven databases and so on

## Additional Information and Declarations

### Data Availability

The following information was supplied regarding data availability: All raw data is available at NCBI SRA: SRR9163842. to SRR9163845.
