# Supplementary figures and images for: Transcriptome profiling of kenaf (Hibiscus cannabinus L.) under plumbic stress conditions implies the involvement of NAC transcription factors regulating reactive oxygen species-dependent programmed cell death
Source: PeerJ. 2020 Mar 10;8:e8733. doi: 10.7717/peerj.8733 (PMC7069409; doi:10.7717/peerj.8733)

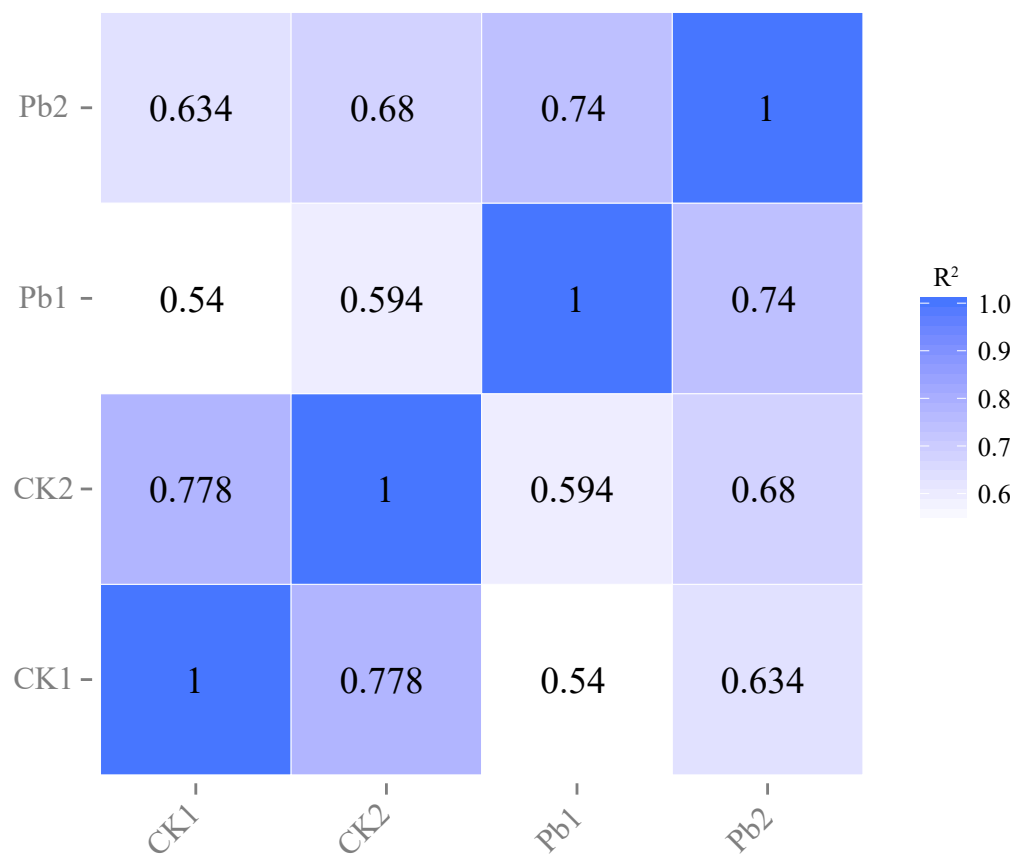

Supplement: Figure S1 [file peerj-08-8733-s001.pdf]

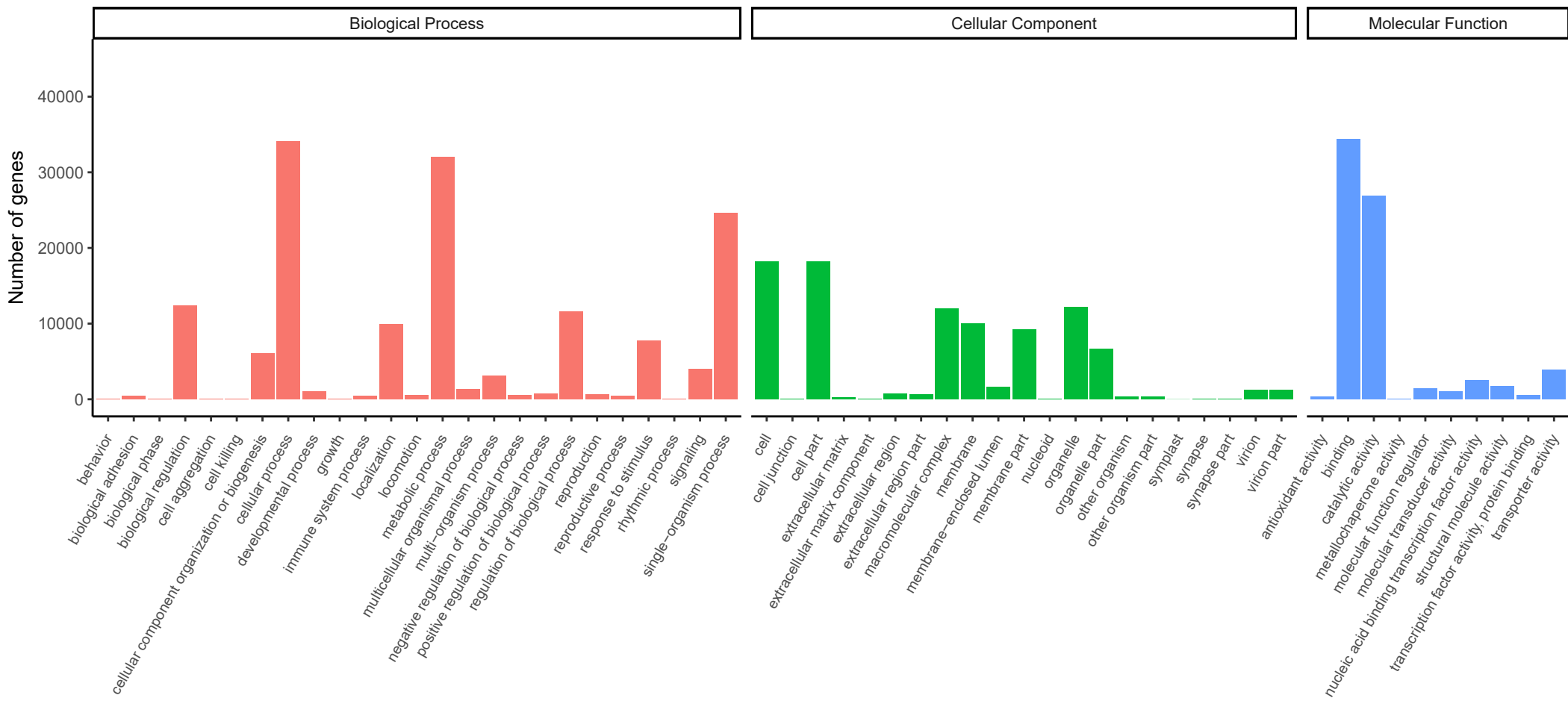

Supplement: Figure S2 [file peerj-08-8733-s002.pdf]

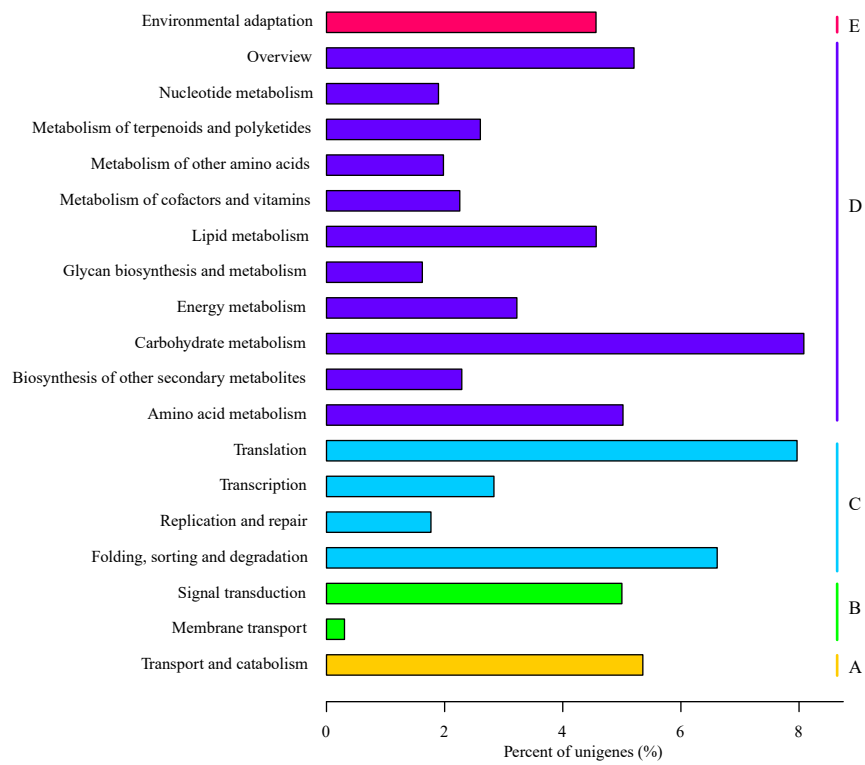

Supplement: Figure S3 [file peerj-08-8733-s003.pdf]

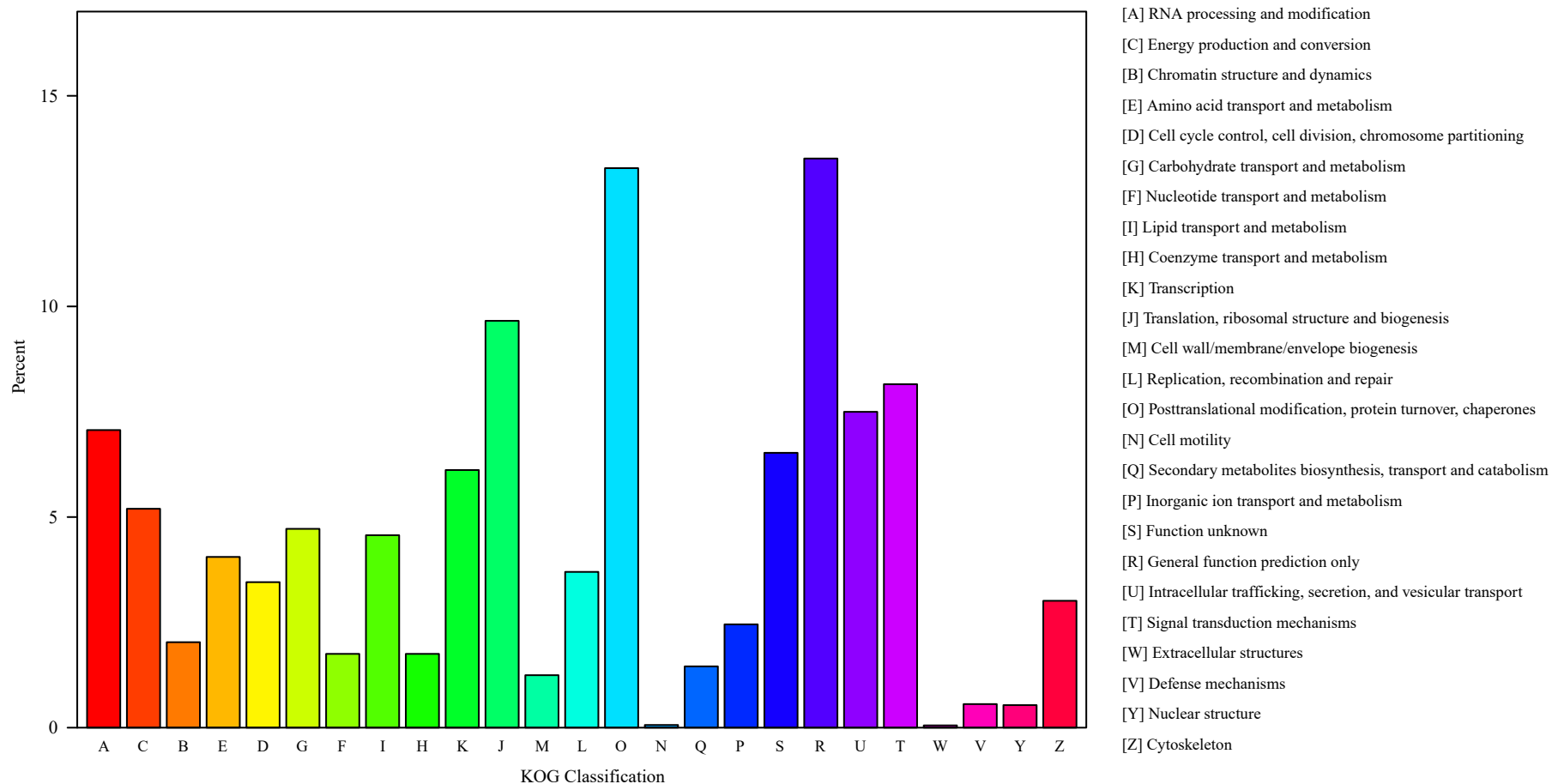

Supplement: Figure S4 [file peerj-08-8733-s004.pdf]

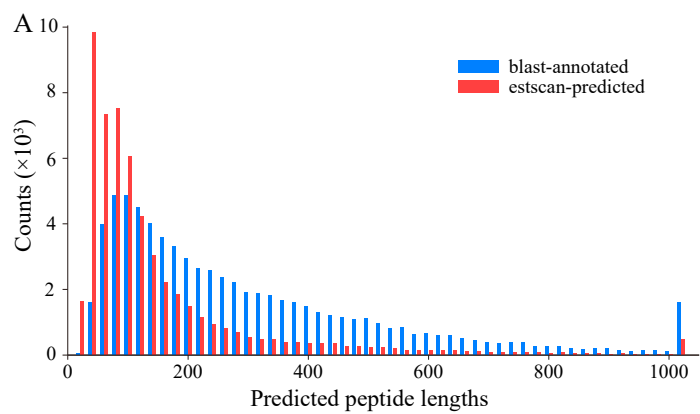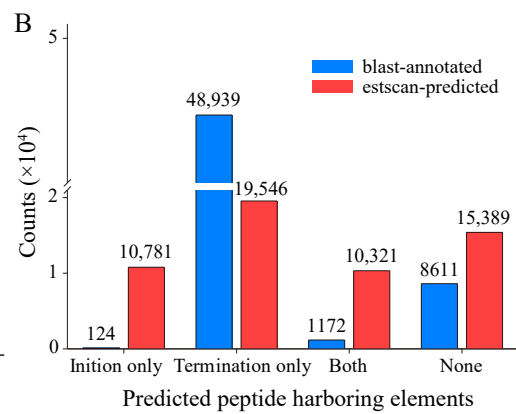

Supplement: Figure S5 — Length distribution of the peptides encoded by unigenes annotated via a BLAST search of the Nr database (blue) and the peptides encoded in unigenes whose open reading frame was predicted with ESTScan (red) (A). The last blue and red columns include peptides longer than 1,000 amino acids. Analyses of the presence of initiation and/or stop codons in the unigenes encoding the peptides in the blue and red columns (B). [file peerj-08-8733-s005.pdf]

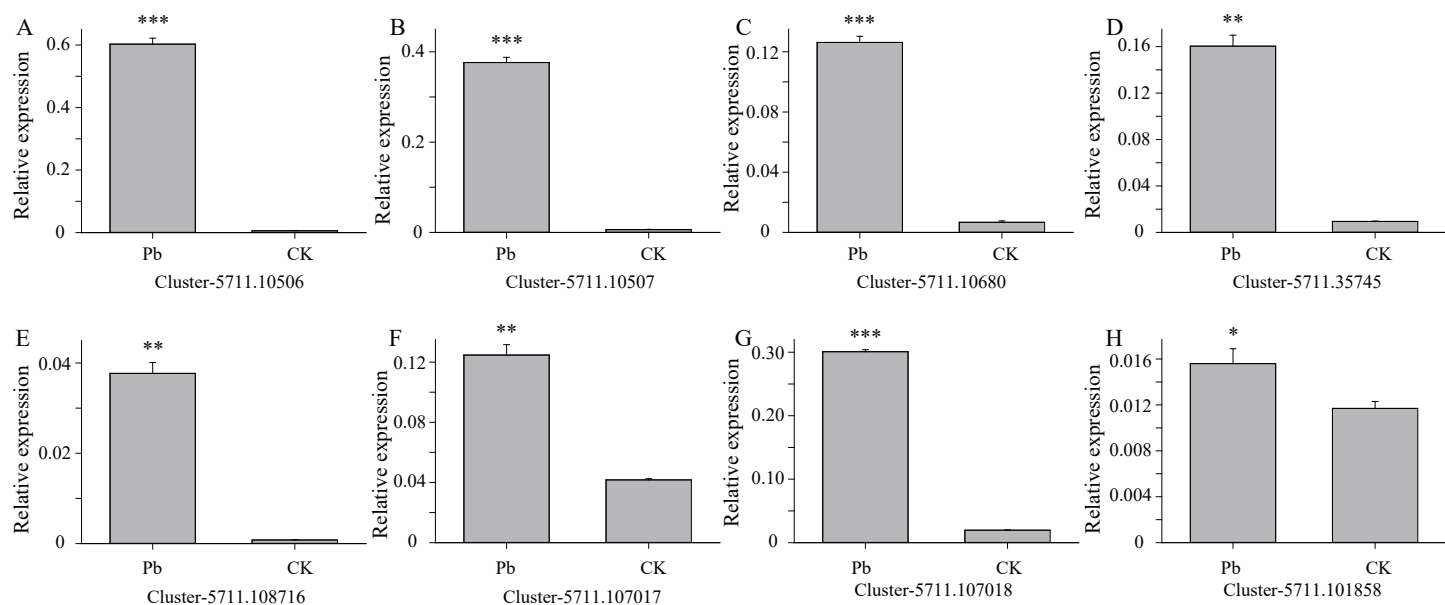

Supplement: Figure S6 — Eight unigenes encoding transcription factors (TFs) were randomly selected, including five NAC TF genes (A–E, corresponding to Cluster-5711.10506, Cluster-5711.10507, Cluster-5711.10680, Cluster-5711.108716, and Cluster-5711.35745, respectively), two ERF genes (F and G, corresponding to Cluster-5711.107017 and Cluster-5711.107018, respectively), and one TCP gene (H, Cluster-5711.101858). Error bars represent the standard deviation. Significant differences between the CK and Pb-stressed samples (as determined with Student’s t-test) are indicated as follows: * p < 0.05, ** p < 0.01, and *** p < 0.001. [file peerj-08-8733-s006.pdf]
